# The Role of IL-6 and TNF-Alpha Biomarkers in Predicting Disability Outcomes in Acute Ischemic Stroke Patients

**DOI:** 10.3390/life15010047

**Published:** 2025-01-02

**Authors:** Ciprian-Ionuț Băcilă, Maria-Gabriela Vlădoiu, Mădălina Văleanu, Doru-Florian-Cornel Moga, Pia-Manuela Pumnea

**Affiliations:** 1 Faculty of Medicine, Lucian Blaga University of Sibiu, Izvorului Street, 550169 Sibiu, Romania; bacila_c@yahoo.com (C.-I.B.); manuelapumnea@yahoo.com (P.-M.P.); 2 Collective of Scientific Research in Neurosciences of the Clinical Psychiatry Hospital “Dr. Gheorghe Preda”, 550082 Sibiu, Romania; 3 Neurology Department, Emergency County Clinical Hospital Sibiu, Corneliu Coposu bvd, 550245 Sibiu, Romania; 4 Department of Medical Informatics and Biostatistics, University of Medicine and Pharmacy “Iuliu Hatieganu” Cluj-Napoca, Victor Babeş Street, No. 8, 400012 Cluj-Napoca, Romania; madalina.valeanu@gmail.com; 5 Clinical Department of Surgery, Military Clinical Emergency Hospital Sibiu, Victoriei Bvd, 550024 Sibiu, Romania; 6 Medlife Hospital, Constituției Street, 550253 Sibiu, Romania

**Keywords:** stroke, inflammation, biomarkers, disability, rehabilitation

## Abstract

Introduction: Inflammatory biomarkers, including Interleukin-6 (IL-6) and Tumor Necrosis Factor-alpha (TNF-alpha), play a significant role in influencing stroke outcomes, particularly in the progression of post-stroke disability. While numerous studies have suggested a correlation between elevated levels of these cytokines and poor functional recovery, further investigation is needed to understand their prognostic value in acute ischemic stroke. Materials and Methods: We conducted a prospective study on 56 patients diagnosed with acute ischemic stroke, evaluating IL-6 and TNF-alpha levels on days 1 and 7 post symptom onset. Stroke severity was assessed using the National Institutes of Health Stroke Scale (NIHSS) and functional outcomes were measured with the modified Rankin Scale (mRS). Statistical analyses were performed to evaluate the association between biomarker levels and stroke severity and recovery. Results: Our study demonstrated that elevated levels of IL-6 and TNF-alpha on both days 1 and 7 were significantly correlated with greater stroke severity and poorer functional outcomes, as indicated by higher NIHSS and mRS scores. These findings are consistent with broader research indicating strong associations between inflammatory cytokines and post-stroke disability, further reinforcing their relevance as prognostic indicators. Conclusions: IL-6 and TNF-alpha are promising biomarkers for predicting stroke severity and functional recovery in acute ischemic stroke. Monitoring these cytokines in the early stages of stroke could aid in identifying patients at higher risk for long-term disability, potentially guiding personalized therapeutic strategies. Further research into anti-inflammatory therapies targeting these cytokines may improve stroke rehabilitation and outcomes.

## 1. Introduction

Ischemic stroke is a leading cause of death and long-term disability globally, resulting from the interruption of blood flow to the brain, which leads to significant neuronal injury and a robust inflammatory response. The intricate pathophysiological mechanisms underlying ischemic stroke involve a complex interplay of cellular and molecular processes, where inflammation emerges as a pivotal contributor to the exacerbation of brain damage [1]. Among the various inflammatory mediators involved, Interleukin-6 (IL-6) and Tumor Necrosis Factor-alpha (TNF-alpha) have garnered considerable attention due to their crucial roles in the inflammatory cascade following ischemic events. IL-6, a multifunctional cytokine, is rapidly upregulated in response to ischemic conditions. It possesses dual characteristics, exhibiting both neuroprotective and neurotoxic effects depending on its concentration and the context of its expression. This cytokine not only modulates immune responses but also influences neuronal survival, making it integral to understanding stroke outcomes [2,3]. Conversely, TNF-alpha serves as a prominent pro-inflammatory mediator that intensifies neuronal injury through mechanisms such as apoptosis, the promotion of inflammatory cell infiltration, and disruption of the blood–brain barrier. Elevated levels of TNF-alpha post-stroke have been consistently associated with unfavorable clinical outcomes, emphasizing its potential as a target for therapeutic intervention [4]. Recent studies have also highlighted the dynamic interplay between IL-6 and TNF-alpha, suggesting that the balance between these cytokines may significantly affect the inflammatory environment in the post-stroke brain [5]. Understanding these complex interactions is critical for developing targeted therapies that aim to modulate inflammation, thereby improving recovery trajectories and minimizing long-term disabilities in stroke survivors. Enhanced knowledge of the roles played by IL-6 and TNF-alpha may lead to novel therapeutic strategies that can optimize recovery and improve the overall quality of life for individuals affected by ischemic stroke [6,7]. Our study aims to synthesize current knowledge on the roles of IL-6 and TNF-alpha in ischemic stroke, explore their potential as biomarkers for disease progression and prognosis, and correlate the degree of disability in ischemic stroke patients with plasma concentrations of these two biomarkers. By delving into the molecular underpinnings of IL-6 and TNF-alpha involvement in ischemic stroke, we hope to uncover novel insights that could pave the way for more effective and targeted interventions in stroke management.

## 2. Materials and Methods

a. Study design

This study was conducted over a 12-month period, from 1 January to 31 December 2021, at the Neurology Clinic of Sibiu County Emergency Hospital. Participants were patients with acute ischemic stroke, admitted within 24 h of symptom onset. All patients consented to participate by signing informed consent forms.

**Figure 1:**
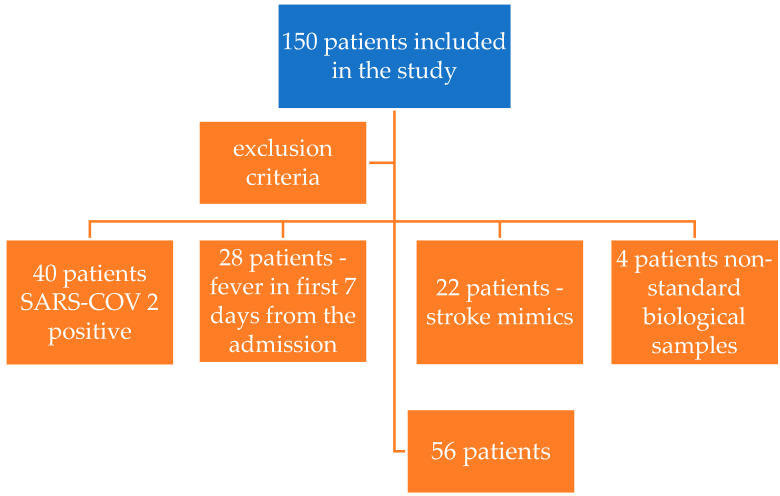
Description of the patients enrolled and excluded in the study.

Initially, 150 patients were included. Of these, 94 were excluded: 40 tested positive for SARS-CoV-2, 28 developed fever within the first 7 days, preventing the second biomarker collection, 22 were diagnosed with stroke mimics, and 4 had non-compliant samples. The final cohort consisted of 56 patients (Figure 1). The patients included in the study were consecutively admitted to the Neurology Department. All participants provided informed consent to participate and met the inclusion criteria. Patients who failed to meet the specified inclusion criteria or presented one or more exclusion criteria were not considered for the study (Table 1).

Blood samples for biomarker analysis were collected within the first 24 h of symptom onset (Day 1) and again on Day 7. The first sample was obtained upon the patient’s arrival at the emergency department during the initial venous access procedure. The median time from symptom onset to sample collection was 10 h. The second biomarker collection (Day 7) was conducted at the same time for all patients, specifically at 7 a.m.

The inclusion and exclusion criteria ensured a focused patient cohort, minimizing confounding factors that could affect the inflammatory biomarker levels.

b. Collection of Biological Samples and Biomarker Measurement

All biological samples were collected in EDTA-coated vacutainers, which were subsequently centrifuged at 1500× *g* for 15 min to separate the plasma. The resulting supernatant was stored at −80 °C to preserve the integrity of the samples. The biomarkers interleukin-6 (IL-6) and tumor necrosis factor-alpha (TNF-alpha) levels were quantified from these samples. Non-standard samples were systematically excluded from the analysis to ensure the validity of the results. Quantification of human IL-6 and TNF-alpha was performed using an enzyme-linked immunosorbent assay (ELISA), employing a quantitative sandwich enzyme immunoassay technique for enhanced specificity and sensitivity.

c. Statistical Analysis

The database was compiled using Microsoft Office Excel 2016, and statistical analysis was conducted with SPSS 25.0 (SPSS Inc., Chicago, IL, USA). The normality of quantitative data distributions was tested using the Shapiro–Wilk or Kolmogorov–Smirnov tests, with an accepted error threshold of α = 0.05. For normally distributed continuous quantitative data, results were expressed as the arithmetic mean ± standard deviation. For non-normally distributed data, the median (quartile 1–quartile 3) was used. Qualitative data were described using frequencies. To compare means of quantitative variables between two independent groups, Student’s t-test was used for normally distributed variables. For variables with non-normal distributions, the Mann–Whitney and Kruskal–Wallis tests were used. A two-way repeated measures ANOVA was used to evaluate changes in IL-6 and TNF-alpha levels over time, with Tukey’s HSD post hoc tests used for pairwise comparisons. Correlation analyses were performed using the Pearson linear correlation coefficient for normally distributed data and the Spearman correlation coefficient for non-normally distributed or ordinal data. Colton’s empirical rules were utilized to interpret the correlation coefficients. The calculated effect sizes in this study (Cohen’s d) ranged in magnitude from 0.65 (moderate effect) to 0.92 (large effect), indicating substantial differences in biomarker levels across time points and outcome groups.

## 3. Results

A total of 56 patients (mean age = 74 years; range = 33–94 years, 50% male) completed the study. The baseline characteristics of the study population, including age, genre, NIHSS scores at admission, IL-6 and TNF-alpha levels, are summarized in Table 2. At baseline, the mean NIHSS score was 14, indicating a moderate to severe stroke population (Table 2).

Of the 56 patients included in the study, 52 (93%) had a baseline modified Rankin Scale (mRS) score between 0 and 2, indicating they were functionally independent or minimally impaired before the ischemic stroke event (Table 3).

Upon discharge, a notable shift in disability was observed: 10% of patients had an mRS score of 3, while 44% had scores between 4 and 5, indicating severe disability for over half of the cohort (Table 3).

A two-way repeated measures ANOVA was conducted to evaluate differences in the levels of IL-6 and TNF-alpha biomarkers across two time points (primary and secondary) in relation to NIHSS scores. The analysis revealed a significant main effect of biomarker type (F(1.55) = 56.951, *p* < 0.001), indicating that IL-6 and TNF-alpha levels differ significantly overall. Additionally, a significant main effect of time (F(1.55) = 18.926, *p* = 0.0001) was observed, demonstrating that biomarker levels varied between the primary and secondary collection points. However, there was no significant interaction effect between biomarker type and time (F(1.55) = 2.766, *p* = 0.103). Post hoc Tukey tests confirmed significant pairwise differences in biomarker levels between conditions. The effect size analysis revealed a moderate to large difference between IL-6 and TNF-alpha levels, with Cohen’s d = −0.92 at the primary collection and d = −0.65 at the secondary collection, indicating that TNF-alpha levels were consistently higher than IL-6 levels at both time points.

The NIHSS scores showed a significant difference between Day 1 and Day 7, as indicated by the two-way repeated measures ANOVA (*p* < 0.001). At Day 1, the mean NIHSS score was 14, and at Day 7, the mean NIHSS score was significantly reduced to 10.2 (*p* = 0.01, Tukey’s post hoc).

**Figure 2:**
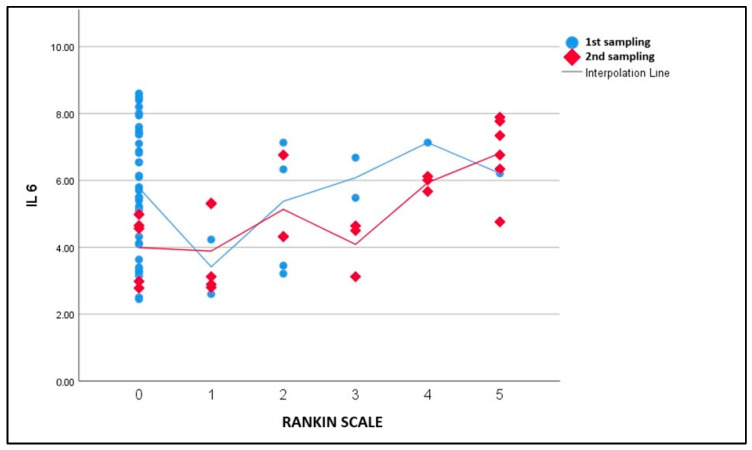
IL-6 levels at Day 1 and Day 7 by Rankin Scale scores.

For IL-6 levels, a significant decrease in mean levels from Day 1 to Day 7 was observed (*p* < 0.001). The IL-6 levels at Day 1 were significantly higher than those at Day 7 (Day 1: 5.47 pg/mL; Day 7: 4.91 pg/mL). There were patients that had increased results (mean increase 30%) (*p* = 0.01, Tukey’s post hoc) (Figure 2). This increase in IL-6 levels was associated with poorer functional recovery as reflected by the NIHSS score.

**Figure 3:**
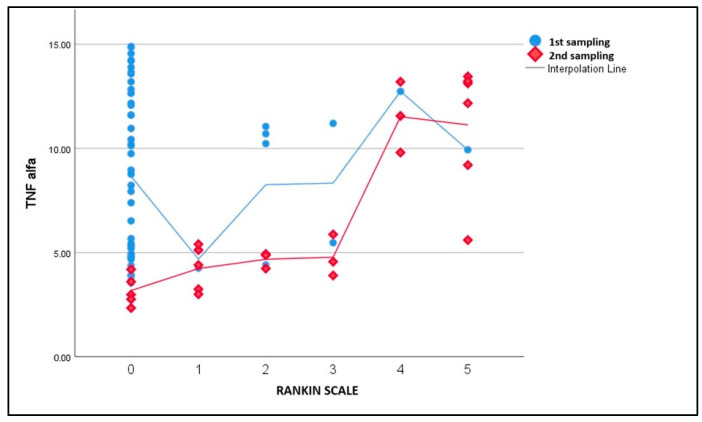
TNF-alpha levels at Day 1 and Day 7 by Rankin Scale scores.

Similarly, TNF-alpha levels demonstrated a significant decrease from Day 1 to Day 7 (*p* < 0.05). At Day 1, the mean TNF-alpha level was 7.39 pg/mL, and by Day 7, it had decreased to 5.12 pg/mL (*p* = 0.01, Tukey’s post hoc) (Figure 3). The higher TNF-alpha levels at Day 7 were also correlated with greater NIHSS scores.

The Rankin score at discharge was significantly correlated with both NIHSS scores and biomarker levels. At discharge, the mean Rankin score was 3.1. A significant positive correlation was found between Rankin score and NIHSS score (*p* < 0.05), as well as with IL-6 (*p* < 0.05) and TNF-alpha (*p* < 0.05) levels.

**Figure 4:**
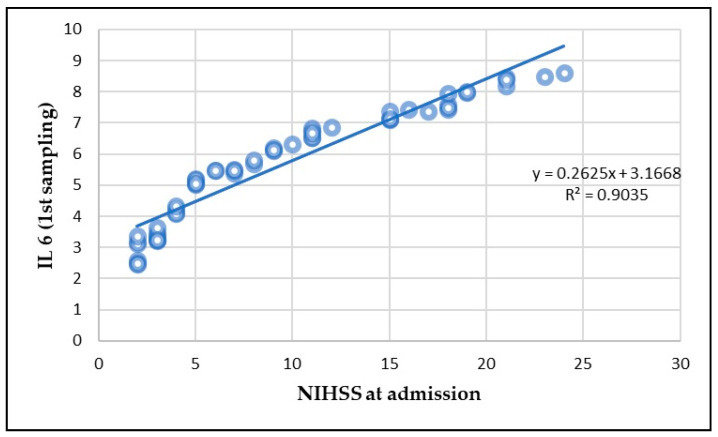
Correlation between IL-6 and NIHSS at admission.

**Figure 5:**
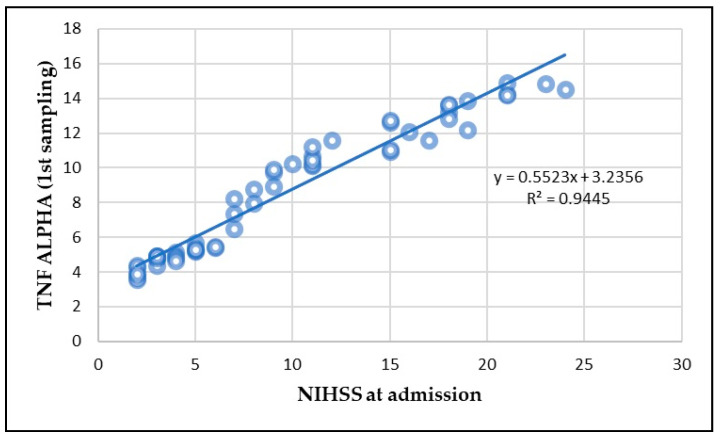
Correlation between TNF-alpha and NIHSS at admission.

A significant correlation between IL-6 and TNF-alpha levels and the NIHSS score was observed. At Day 1, the correlation between IL-6 and NIHSS was strong (*p* < 0.01), indicating that higher IL-6 levels were associated with higher stroke severity (Figure 4). A similar correlation was found for TNF-alpha (*p* < 0.01) (Figure 5). 

A positive correlation was observed between cytokine levels (IL-6 and TNF-alpha) and modified Rankin Scale (mRS) scores at discharge. Spearman’s correlation coefficient demonstrated a significant direct relationship between the two biomarkers and the modified Rankin Scale (Table 4).

**Figure 6:**
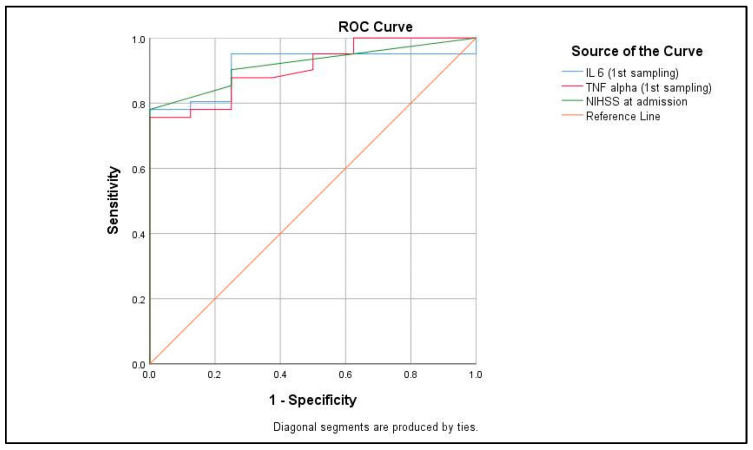
Predictive value of ROC curve in assessing stroke outcome and disability.

Receiver operating characteristic (ROC) analysis was performed to assess the diagnostic accuracy of IL-6, TNF-alpha, and NIHSS at admission in predicting stroke outcomes (Figure 6). The area under the curve (AUC) for IL-6 at the first sampling was 0.912 (95% CI: 0.826–0.997), indicating excellent discriminatory ability. TNF-alpha at the first sampling showed an AUC of 0.907 (95% CI: 0.819–0.995). The NIHSS score at admission exhibited the highest AUC of 0.918 (95% CI: 0.839–0.996), confirming its strong performance in distinguishing between moderate (NIHSS < 15) and severe (NIHSS ≥ 15) stroke (Table 5). All three biomarkers demonstrated statistically significant results (*p* < 0.001).

Throughout the study, six patients died. However, these deaths did not impact the statistical analysis, as they occurred more than seven days after hospital admission, following the second biomarker collection.

## 4. Discussion

Recent studies have significantly advanced our understanding of the role of inflammation, particularly through the involvement of the cytokines Interleukin-6 (IL-6) and Tumor Necrosis Factor-alpha (TNF-alpha), in influencing disability outcomes following acute ischemic stroke. Elevated levels of IL-6 and TNF-alpha during the acute phase of ischemic stroke have been strongly associated with more severe initial disability [7]. These pro-inflammatory cytokines contribute to neuroinflammation, blood–brain barrier disruption, and subsequent neuronal injury, all of which exacerbate stroke-related disability. Emerging evidence suggests that persistent inflammation, indicated by sustained high levels of these cytokines, correlates with worse long-term functional outcomes and increased disability [8].

Zhai et al. conducted a study involving 100 patients diagnosed with acute ischemic stroke, concluding that elevated IL-6 and TNF-alpha levels at the time of admission were significantly associated with poorer functional outcomes and greater disability at discharge. Their research indicated that higher levels of these cytokines correlate with increased stroke severity and poorer recovery outcomes, as measured by standardized scales like the National Institutes of Health Stroke Scale (NIHSS) and the modified Rankin Scale (mRS) [9,10]. This suggests that measuring these cytokines could serve as a useful prognostic tool in clinical settings.

Similarly, Liu et al. conducted a multi-center study involving 350 patients that reinforced the notion that IL-6 and TNF-alpha are significant predictors of both immediate and long-term disability. Their findings indicate that the inflammatory response measured during the acute phase of stroke can reliably forecast recovery trajectories [11,12]. Such insights are critical, as they allow clinicians to identify patients at higher risk for long-term disability, enabling more targeted therapeutic interventions.

Patel et al. also support the conclusion that higher levels of IL-6 and TNF-alpha early in the post-stroke period are linked to greater long-term disability and slower recovery. Their study conducted on 200 patients with acute ischemic stroke demonstrates a clear correlation between sustained inflammation and neurodegeneration, indicating that effective management of these cytokines could have a substantial impact on patient outcomes [13]. Additionally, they highlight the importance of monitoring cytokine levels during hospitalization to better predict recovery patterns [14].

The patients included in our research demonstrated significant correlations between the biomarkers IL-6 and TNF-alpha and the modified Rankin Scale, highlighting the crucial role of inflammation in the disability prognosis of stroke patients. Significantly elevated plasma levels of inflammatory biomarkers were correlated with moderate-to-severe disability and an unfavorable prognosis in these patients.

A systematic review and meta-analysis by Zhang et al. examined the association between circulating inflammatory markers, including IL-6 and TNF-alpha, and functional outcomes after acute ischemic stroke. This comprehensive review found that elevated levels of these cytokines were consistently associated with poorer functional outcomes and increased disability, reinforcing their potential role as prognostic biomarkers in stroke recovery. The inclusion of various studies in this meta-analysis adds strength to the argument for the significance of these inflammatory markers in understanding stroke prognosis [15].

In a longitudinal study by Tzeng et al., researchers tracked inflammatory markers in a cohort of 150 patients over six months post-stroke. They found that patients with persistently elevated IL-6 and TNF-alpha levels were more likely to experience significant cognitive decline in addition to physical disability. This highlights, in accordance with Kim et al., the dual impact of inflammation on both cognitive and physical recovery, suggesting that therapeutic strategies aimed at reducing inflammation could benefit multiple aspects of stroke recovery [16,17].

Rodriguez et al. further support the importance of managing inflammation, noting that patients with higher initial levels of IL-6 not only exhibited poorer recovery outcomes but also had an increased risk of recurrent strokes within a year. Their findings underscore the necessity for ongoing monitoring and intervention aimed at modulating inflammatory responses in stroke patients, as these factors may significantly influence long-term health outcomes [18].

Similarly, the relationship between inflammation, specifically through cytokines such as IL-6 and TNF-alpha, and stroke severity has been extensively studied. Elevated levels of these cytokines have been linked to more severe neurological deficits, as measured by tools such as the NIHSS [19,20]. In our study, the direct proportional correlation between IL-6, TNF-alpha, and NIHSS scores further emphasizes the role of these inflammatory markers in the early and late stages of ischemic stroke. Elevated IL-6 and TNF-alpha levels during the acute phase of stroke have been shown to exacerbate neuronal injury through various mechanisms, including apoptosis and disruption of the blood–brain barrier.

Similar findings were observed in studies by Zhai et al., who found that elevated IL-6 and TNF-alpha levels at stroke onset were associated with greater disability as measured by NIHSS and the modified Rankin Scale (mRS). Their research suggested that the inflammatory response plays a key role in exacerbating stroke severity and can help predict functional recovery [9]. Additionally, Liu et al. reinforced these findings in a multi-center study, highlighting the potential of IL-6 and TNF-alpha as reliable predictors of post-stroke disability. Elevated cytokine levels correlated with both acute stroke severity and long-term disability, underscoring the importance of inflammation as a therapeutic target [21].

In line with this, our findings provide additional evidence that supports the notion of cytokine-driven neuroinflammation playing a crucial role in stroke outcomes. The positive correlation between IL-6, TNF-alpha, and NIHSS scores in our study suggests that these biomarkers are not only indicators of stroke severity but may also be utilized in the early prediction of functional outcomes. In agreement with previous studies, such as those conducted by Patel et al. and Zhang et al., sustained inflammation is associated with worse long-term outcomes and increased disability. This relationship underscores the need for timely inflammatory management in acute stroke patients to improve recovery trajectories [14,22].

In all patients, biomarker levels, particularly IL-6 and TNF-alpha, were elevated at both time points. However, a decline in biomarker levels was observed in those patients whose neurological function improved, reflected by a lower NIHSS score at discharge compared to admission. Conversely, patients whose condition worsened or had poor clinical outcomes during hospitalization demonstrated an increase in biomarker levels over the 7-day period. This differential pattern suggests a potential correlation between biomarker fluctuations and stroke recovery trajectories.

Similarly to recent research, in our study, biomarker levels, particularly IL-6 and TNF-alpha, were elevated at both time points. However, a decline in these levels was observed in patients with improved neurological function, as indicated by a lower NIHSS score on Day 7 compared to admission. Conversely, patients with worsening conditions or poor clinical outcomes during hospitalization exhibited an increase in biomarker levels over the 7-day period. This differential pattern underscores a potential association between biomarker fluctuations and stroke recovery trajectories.

Further studies have also explored the role of these cytokines in post-stroke rehabilitation. Research by Okada et al. found that patients with higher levels of TNF-alpha at the start of rehabilitation exhibited slower recovery. This suggests that managing inflammatory cytokines during the post-stroke rehabilitation phase may improve recovery outcomes [23]. Additionally, Lee et al. and Yang et al. explored anti-inflammatory strategies targeting IL-6 and TNF-alpha in stroke patients, demonstrating that these approaches, when combined with standard rehabilitation therapies, led to better functional outcomes and reduced disability [24,25].

Given the increasing recognition of inflammation’s role in stroke recovery, there is a growing interest in therapeutic interventions that target inflammatory pathways. The use of anti-inflammatory agents to modulate IL-6 and TNF-alpha levels in stroke patients is currently under investigation [26]. Preliminary results from studies like those by Yang et al. suggest that targeting these cytokines may significantly improve functional recovery and reduce post-stroke disability [17,27].

Our study also reinforces the importance of early monitoring of cytokine levels, as persistent elevation of IL-6 and TNF-alpha can indicate a higher likelihood of poor recovery. Serial cytokine measurements could be invaluable in clinical practice, providing early prognostic information that can guide therapeutic decision-making. Moreover, combining this biomarker data with clinical assessments like the NIHSS could enhance the precision of outcome predictions, allowing for more individualized treatment plans.

Novel therapeutic approaches are currently exploring the use of anti-inflammatory treatments to mitigate post-stroke disability. Lee et al. evaluated the effects of anti-inflammatory therapies targeting IL-6 and TNF-alpha during stroke rehabilitation. Their study found that patients receiving these therapies alongside standard rehabilitation demonstrated improved functional outcomes and reduced disability compared to those receiving standard care alone. This emphasizes the potential of targeting specific inflammatory pathways in enhancing recovery [28,29].

Similarly, Yang et al. explored the therapeutic benefits of targeting IL-6 and TNF-alpha with anti-inflammatory treatments, reporting that patients who received such treatments exhibited better functional recovery and lower levels of disability than those who only received conventional care. Their findings suggest that addressing inflammation could enhance rehabilitation efforts and improve recovery outcomes [30]. These studies indicate a shift in focus toward incorporating anti-inflammatory strategies into standard post-stroke care protocols.

The interaction between inflammation and rehabilitation therapies is an area of growing interest. Research indicates that high levels of IL-6 and TNF-alpha could negatively impact the effectiveness of rehabilitation interventions. For instance, a study by Okada et al. demonstrated that patients with elevated levels of TNF-alpha at the start of rehabilitation had slower rates of functional recovery [31]. This highlights the need for integrated approaches that combine anti-inflammatory strategies with conventional rehabilitation, as the timing of rehabilitation interventions relative to cytokine levels may be crucial. Early rehabilitation, in conjunction with anti-inflammatory treatment, could optimize recovery trajectories [32].

As we move forward, the exploration of novel anti-inflammatory treatments targeting IL-6 and TNF-alpha presents promising avenues for improving post-stroke recovery and reducing long-term disability. Integrating these therapeutic strategies with conventional rehabilitation approaches may optimize recovery, highlighting the necessity for a multidisciplinary approach in stroke care. Furthermore, understanding the individual variability in inflammatory responses and utilizing advanced imaging techniques could enhance our ability to predict outcomes and tailor interventions effectively [33,34].

Emerging treatments targeting IL-6 and TNF-alpha present promising advances in post-stroke care. Anti-inflammatory therapies, when integrated with standard rehabilitation, have demonstrated improved recovery and reduced disability by mitigating neuroinflammation [35]. Studies highlight the benefits of early, personalized interventions, leveraging inflammatory biomarker profiles to optimize therapy outcomes. Techniques such as cytokine modulation not only enhance functional recovery but also reduce the risk of recurrent strokes and cognitive decline. Combining these treatments with innovative rehabilitation strategies underscores the evolving therapeutic potential in addressing both immediate and long-term consequences of ischemic stroke [36,37].

As our understanding of the inflammatory processes involved in stroke continues to evolve, future research should aim to elucidate the precise mechanisms through which inflammation impacts both cognitive and physical recovery [38]. Identifying specific inflammatory biomarkers could pave the way for personalized treatment approaches that not only address inflammation but also enhance neuroplasticity, the brain’s ability to reorganize and adapt post-injury [39].

Limitations of the study:

(a) Sample size: While the study included 56 patients, a larger cohort may be necessary to generalize the findings across diverse populations and settings;
(b) Timing of biomarker measurement: Biomarkers were measured only on days 1 and 7, which may miss important fluctuations in cytokine levels during the acute and subacute phases of stroke;
(c) Single-center study: The study was conducted at a single institution. This may limit the generalizability of the findings to other centers with different patient populations or clinical practices;
(d) Lack of long-term follow-up: The study may not account for the long-term evolution of inflammation and its effects on stroke recovery, focusing primarily on early post-stroke outcomes;
(e) Exclusion of other biomarkers: The study focused on IL-6 and TNF-alpha, but there are other inflammatory and non-inflammatory markers that may also play a role in stroke prognosis, potentially limiting the scope of the analysis;
(f) Measurement techniques: Variations in the sensitivity or specificity of assays used to measure IL-6 and TNF-alpha might affect the reliability of biomarker levels;
(g) Heterogeneity in stroke severity: Patients with varying degrees of stroke severity may exhibit different inflammatory responses, making it harder to establish clear-cut correlations between cytokine levels and outcomes.

These limitations should be acknowledged when interpreting the results and drawing conclusions about the clinical utility of IL-6 and TNF-alpha as prognostic biomarkers.

## 5. Conclusions

This study underscores the critical role of inflammatory biomarkers, specifically IL-6 and TNF-alpha, in the prognosis and functional recovery of patients with acute ischemic stroke. The significant correlations observed between these cytokines, stroke severity as measured by NIHSS, and disability outcomes as assessed by the modified Rankin Scale highlight their potential as reliable predictors of both acute and long-term recovery. The dynamic changes in IL-6 and TNF-alpha levels from admission to Day 7 further emphasize the importance of early and continuous monitoring of these biomarkers.

Our findings align with the existing literature, reinforcing the concept that persistent inflammation exacerbates neuronal injury and hinders recovery. The identification of IL-6 and TNF-alpha as key contributors to post-stroke outcomes suggests that therapeutic strategies targeting cytokine modulation could be pivotal in improving rehabilitation trajectories and reducing long-term disability.

## Figures and Tables

**Table 1:** Inclusion and exclusion criteria.

Inclusion Criteria	Exclusion Criteria
Adult patients (aged over 18 years) who were admitted to the Neurology Department of the Sibiu County Emergency Clinical Hospital.	Patients exhibiting any medical conditions that may interfere with inflammatory marker levels, including infections, autoimmune disorders, neoplasms, or hematological conditions such as lymphoma and multiple myeloma.
Patients presenting with neurological symptoms and signs that are strongly indicative of ischemic stroke occurring within the preceding 24 h.	Individuals who received corticosteroid or immunosuppressive treatments within the preceding 30 days.
Cerebral imaging results confirming the absence of cerebral tumors or hemorrhagic stroke.	Patients with a history of acute myocardial infarction, myocarditis, or acute ischemic stroke within the prior 180 days.
	Those presenting with documented traumatic brain injuries at the time of admission.
	Patients that received IV thrombolysis or thrombectomy.

**Table 2:** Characteristics of the patients included in the study.

Variable	Unit of Measurement	Average/Median
Age *	years	74 (70–80)
Gender **	F	28 (50%)
	M	28 (50%)
IL-6 (First Sampling) ***	pg/mL	5.47 (1.72)
IL-6 (Second Sampling) ***	pg/mL	4.91 (1.68)
TNF-Alpha (First Sampling) *	pg/mL	7.39 (4.9–10.96)
TNF-Alpha (Second Sampling) *	pg/mL	5.12 (3.9–9.34)
NIHSS at Admission *	points	14 (11–18)

Notes: (*)—non-normality quantitative variable described using median (first quartile-third quartile); (**)—qualitative variables described using absolute frequency (relative frequency%); (***)—normality quantitative variable described using arithmetic mean (standard deviation).

**Table 3:** Modified Rankin Scale (mRS) scores at admission and discharge in study participants.

Rankin Scale	Points	Number of Patients
Pre-Admission Rankin *	0	45 (80.4%)
	1	2 (3.6%)
	2	5 (8.9%)
	3	2 (3.6%)
	4	1 (1.8%)
	5	1 (1.8%)
Rankin at Discharge *	0	6 (12%)
	1	9 (18%)
	2	8 (16%)
	3	5 (10%)
	4	11 (22%)
	5	11 (22%)

(*) qualitative variables described using absolute frequency (relative frequency%).

**Table 4:** Spearman coefficients for the two biomarkers included in the study.

Biomarker	First Sampling	Second Sampling	*p*-Value
**IL-6**	0.723 **	0.705 **	<0.001
**TNF-Alpha**	0.719 **	0.823 **	<0.001

Note: (**)—correlation is significant, *p* < 0.001.

**Table 5:** AUC evaluation of IL-6, TNF-alpha, and NIHSS in predicting stroke severity and functional recovery.

Area Under the Curve					
Test Result Variable(s)	Area	Std. Error ^a^	Asymptotic Sig. ^b^	Asymptotic 95% Confidence Interval	
				Lower Bound	Upper Bound
IL-6 (first sampling)	0.912	0.044	0.000	0.826	0.997
TNF-alpha (first sampling)	0.907	0.045	0.000	0.819	0.995
NIHSS at admission	0.918	0.040	0.000	0.839	0.996

^a^ is Under the nonparametric assumption; ^b^ is Null hypothesis: true area = 0.5.

## Data Availability

The data supporting this study are available upon request from the corresponding author. Due to privacy and ethical considerations, the data are not publicly accessible.
